# Research progress on FOXM1 in ovarian cancer diagnosis and therapeutics

**DOI:** 10.3389/fonc.2025.1598868

**Published:** 2025-06-19

**Authors:** Xiao-Qing Tan, Ai-Ying Guo, Li-Fei Zheng, Jun Xiong

**Affiliations:** Department of Obstetrics and Gynecology, The Second Affiliated Hospital of Nanchang University, Nanchang, Jiangxi, China

**Keywords:** ovarian cancer, FOXM1, cancer biomarkers, early detection, cancer diagnosis

## Abstract

Ovarian cancer (OC) is the leading cause of cancer-related death among women, presenting a significant threat to their lives and health. Early-stage OC often lacks distinctive clinical symptoms, leading to most patients being diagnosed at advanced stages. Current treatment strategies primarily involve a combination of surgical resection and chemotherapy, but the therapeutic outcomes are limited, and prognosis remains poor. Therefore, there is a critical need to understand the pathogenesis of OC, identify biomarkers for early diagnosis and prognosis, and discover new therapeutic targets. Forkhead box M1 (FOXM1), recognized as a pro-oncogenic transcription factor (TF), is notably overexpressed in various malignancies, including OC. Research indicates that increased levels of FOXM1 correlate significantly with OC’s aggressive behaviors such as proliferation, invasion, migration, epithelial-mesenchymal transition (EMT), and resistance to chemotherapy. These observations suggest that FOXM1 could potentially function as both a biomarker and a therapeutic target, facilitating the early detection and treatment of OC.

## 1 Introduction

Ovarian cancer (OC) ranks as the third most prevalent gynecological cancer globally and is the deadliest among gynecological tumors, with a five-year survival rate hovering around 48% (1). This poses a significant threat to women’s health and well-being. The absence of distinct clinical symptoms and reliable biological markers makes early detection difficult, resulting in most patients being diagnosed at advanced stages (2, 3). Advanced OC frequently leads to intra-abdominal metastasis, which damages abdominal organs and tissues, contributing to the poor prognosis (4). Currently, the treatment of OC primarily involves a combination of surgical resection and chemotherapy (5). However, the therapeutic effect is limited, often accompanied by chemoresistance, and associated with a high risk of recurrence (6). Thus, there is an urgent need to understand the molecular mechanisms of OC, identify related target genes, and discover novel biomarkers and therapeutic targets. These efforts could significantly improve early diagnosis and treatment outcomes for OC (3).

Initially discovered in mice as Trident, FOXM1 belongs to the FOX family of transcription factors, essential in embryonic development (7–9). It has also been known as HFH-11 (10), FKHL-16 (11), WIN (12), and MPP-2 (13) across different species. FOXM1 orchestrates vital cellular functions including growth, proliferation, differentiation, metabolism, and apoptosis (14). Recent attention has focused on FOXM1 due to its significant overexpression in various human cancers and its crucial role in tumor advancement (15). As a result, FOXM1 has become a promising candidate for the early diagnosis and treatment of OC (8).

In this review, the oncogenic effects of FOXM1 in OC are deeply explored, which enhances understanding of its underlying mechanisms, discusses the relevant challenges of targeting FOXM1, and pays special attention to the latest FOXM1 inhibitors, such as EBT inhibitors and Thiostrepton, and analyzes their potential applications in the clinical context of OC, providing a new perspective for future translational medicine research.

## 2 FOXM1 overview

### 2.1 Structure and isoforms of FOXM1

**Figure 1:**
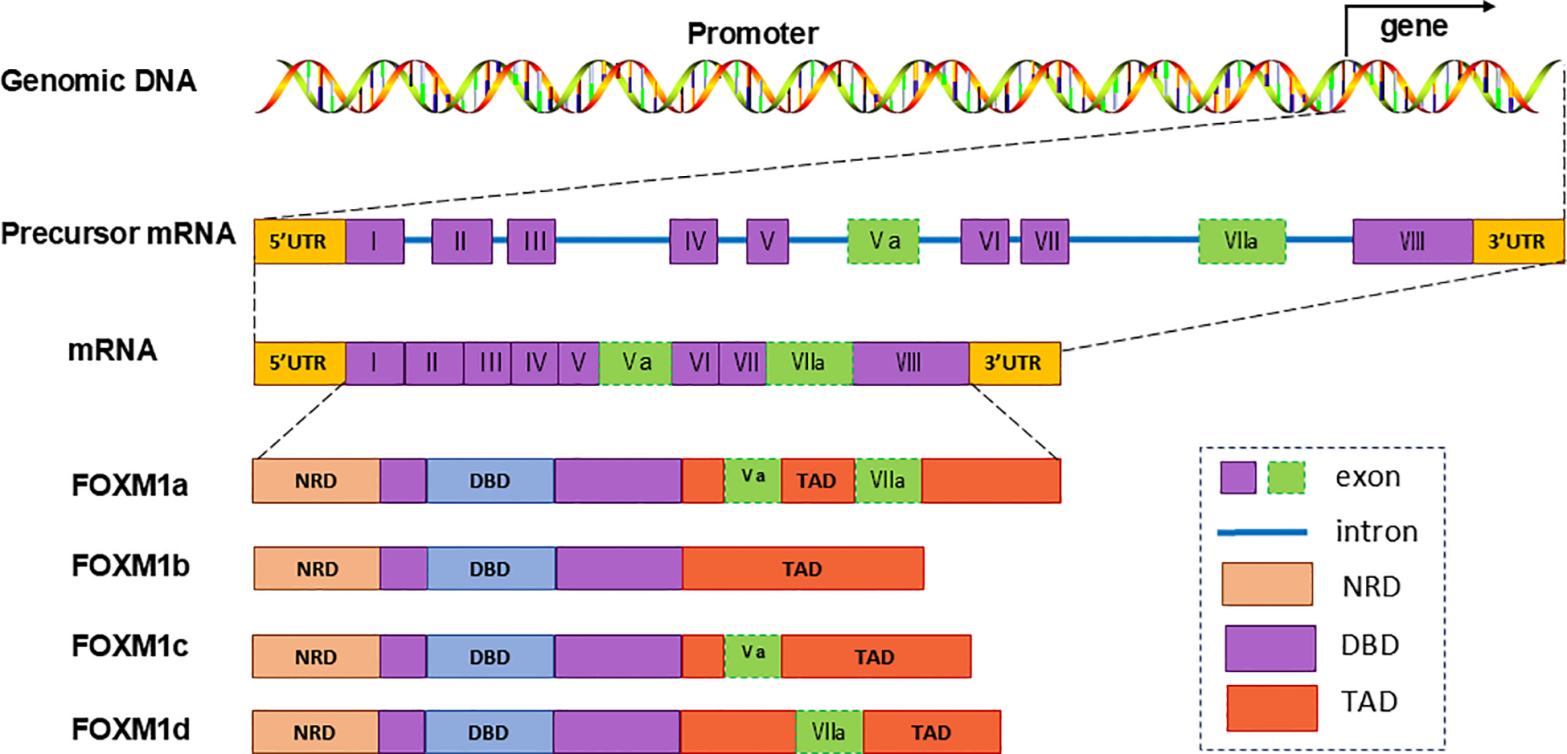
Genomic group structure and splicing isoforms of FOXM1.

TFs within the FOX protein family share a conserved DNA-binding winged helix domain (9, 16) The human FOXM1 gene, comprising 10 exons, is situated on chromosome 12p13.33. Alternative splicing of exons Va and VII leads to the formation of four FOXM1 isoforms: FOXM1a, FOXM1b, FOXM1c, and FOXM1d (8, 16). FOXM1a, incorporating both exons Va and VIIa, loses transcriptional activity due to the insertion of exon VIIa into its activation domain. Conversely, FOXM1b, FOXM1c, and FOXM1d maintain transcriptional activity, each exhibiting distinct functional characteristic (8, 17, 18) (**Figure 1**).

### 2.2 Function and regulation of FOXM1

FOXM1 is a critical TF that regulates cell proliferation and exhibits a cell cycle-specific expression pattern (19). It controls the transcription of several cell cycle-related genes, ensuring accurate DNA replication and mitosis (20). Additionally, FOXM1 regulates various essential biological processes, playing an active role in cell proliferation, migration, angiogenesis, stem cell regeneration, DNA damage repair, apoptosis, and inflammation (14, 21).

The expression of FOXM1 is regulated at multiple levels. Transcriptional regulation of FOXM1 involves several factors, including the CCCTC-binding factor (CTCF) (22), cAMP-responsive element-binding protein (CREB) (23), signal transducer and activator of transcription 3 (STAT3) (24), Twist 1 (25), and E2F (26), which can directly bind to the FOXM1 promoter and enhance its expression. Post-transcriptionally, several miRNAs can regulate FOXM1 by binding to its 3’ UTR, a mechanism observed in many cancers (27–30). Furthermore, FOXM1 undergoes various post-translational modifications (PTMs), including ubiquitination, phosphorylation, methylation, and acetylation (16, 31–33). These PTMs can either activate or inhibit its transcriptional activity, protein stability, and cellular localization (34).

### 2.3 FOXM1 and malignant tumors

As a proto-oncogene, FOXM1 is highly expressed in various human cancers, promoting malignant cell proliferation in tumors such as gastric, breast, lung, pancreatic, colorectal, cervical, and prostate cancers (8, 35–37). The upregulation of FOXM1 enhances the proliferation, migration, and invasive potential of cancer cells (8). Recent research has revealed that the expression of FOXM1 is notably increased in OC tissues as compared to adjacent non-cancerous tissues. This overexpression significantly contributes to the oncogenesis and metastatic spread of OC (38–40). Llauradó et al. found that FOXM1 expression was upregulated in most OC specimens. This was determined by examining FOXM1 expression in 34 OC and 11 normal ovarian specimens. The analysis revealed that FOXM1 expression was closely associated with the stage of OC and the malignant invasive tumor phenotype. The higher the cancer stage, the higher the expression level of FOXM1, and the poorer the prognosis (39). Ning et al. noted a marked elevation in FOXM1 levels within OC samples, particularly pronounced among patients who exhibited lymph node metastasis versus those who did not (40). These observations underscore the pivotal role of FOXM1 TFs in the progression of OC and highlight its potential as an innovative therapeutic target.

## 3 Role of FOXM1 in the development of OC

**Figure 2:**
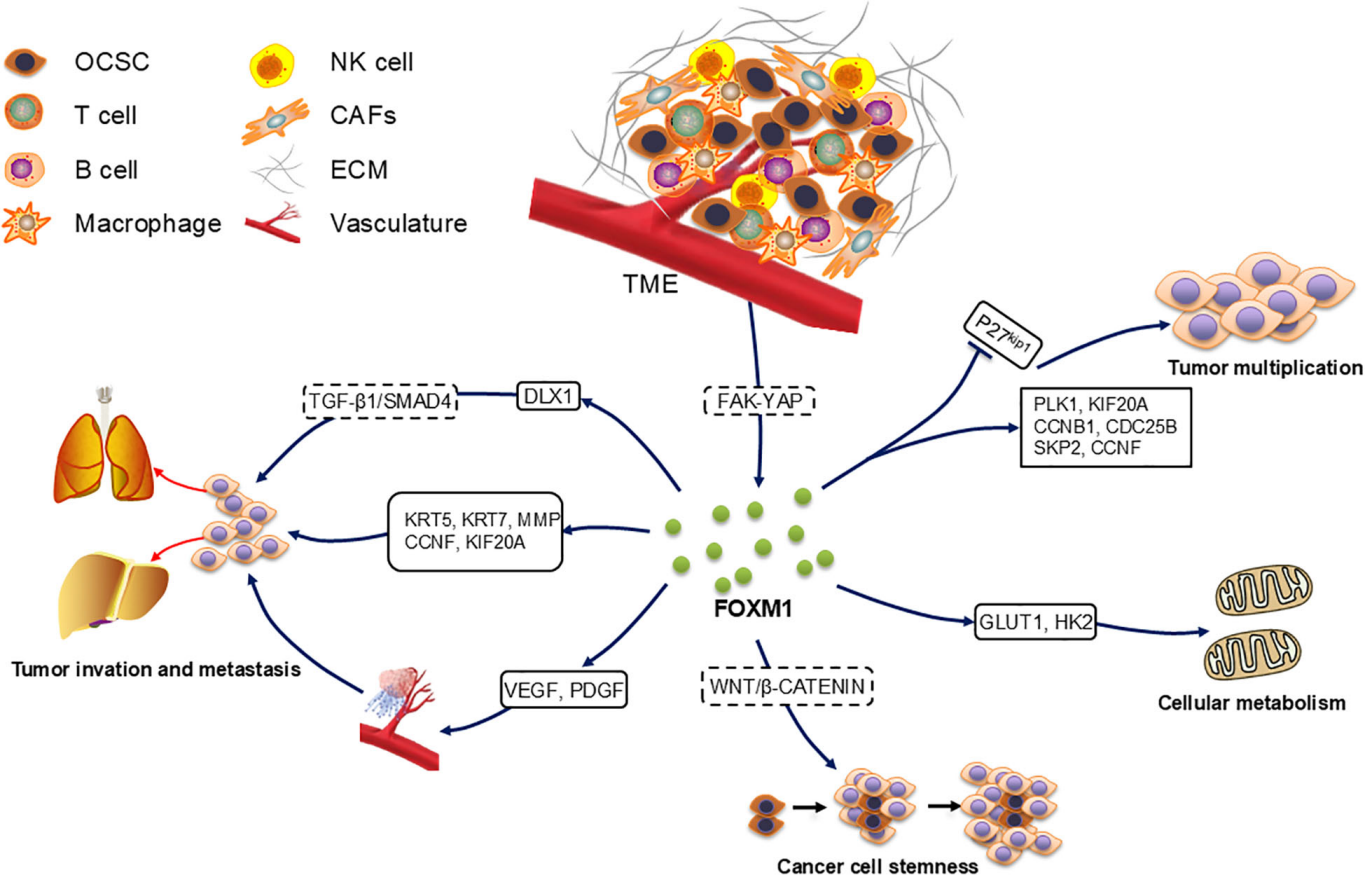
Role of FOXM1 in ovarian cancer development. FOXM1 accelerates the cell cycle and thus promotes cell proliferation by up-regulating the expression of downstream target genes PLK1, KIF20A, CCNB1, CDC25B, SKP2, and CCNF and by accelerating the degradation of p27^KiP1^. OCSC in contact with TME activated the FAK-YAP pathway to increase the expression of FOXM1, which increased cancer stemness through the Wnt/β-catenin pathway. FOXM1 also accelerated cellular metabolic processes through the up-regulation of GLUT1 and HK2.FOXM1 up-regulated VEGF and PDGF to regulate angiogenesis, as well as through the downstream target genes KRT5, KRT7, MMP, CCNF, KIF20A and DLX1 promoted cancer cell metastasis.

FOXM1 upregulation impacts several fundamental tumor biological functions such as cell proliferation, apoptosis regulation, tissue invasion, metastasis, angiogenesis, stem cell properties of tumors, and alterations in metabolic processes (**Figure 2**). FOXM1 exerts its biological effects through various molecular mechanisms, promoting the progression of OC.

### 3.1 FOXM1 promotes OC cell proliferation, invasion and metastasis

In multiple experimental models, FOXM1 promotes tumor cell proliferation by sustaining proliferative signaling and evading growth-inhibitory factors, which enhances cell viability and expedites cell cycle progression. Knockdown of FOXM1 inhibits the expression of cell cycle genes and suppresses cell proliferation, colony formation, and tumor growth. Further studies have confirmed FOXM1’s role in enhancing the proliferation, invasion, and metastasis of OC cells through the modulation of gene expression downstream (**Table 1a**, **Table 1b**).

**Table 1a:** FOXM1 target genes and mechanisms in cell proliferation.

Target genes	Cell model	Expression	Possible mechanisms of carcinogenesis	Significance	Refs
CDC25B	EOC-CC1, OSPC2	Up-regulated	Mediates cell cycle progression	/	(41, 42)
CCNB1	IOSE-SV, COV362, hOSE	Up-regulated	Promotes mitotic progression	/	(43)
PLK1	SKOV3, A2780	Up-regulated	Mediates mitosis and cytoplasmic dissociation	Therapeutic targets	(44, 45)
P27^kip1^	SKOV3, A2780	Down-regulated	Blocks cell cycle progression by inhibiting cyclin-CDK activity	Prognostic marker	(45–47)
SKP2	U_2_OS	Up-regulated	Involved in substrate recognition and degradation of proteins responsible for cell cycle processes	/	(48)
CCNF	A2780, SKOV3, OVCAR3, HEY, HEK293T	Up-regulated	Regulate the stability of proteins involved in the cell cycle and genome stability	Prognostic marker, therapeutic targets	(49)
KIF20A	A2780, SKOV3, OVCAR3, HEY, HEK293T	Up-regulated	Accumulates in mitotic cells, involved in mitosis and chromosome transport	Prognostic marker, therapeutic targets	(49)

**Table 1b:** FOXM1 s target genes and mechanisms in cell invasion and metastasi.

Target genes	Cell model	Expression	Possible mechanisms of carcinogenesis	Significance	Refs
Cell invasion and metastasis					
MMP	/	Up-regulated	Mediates submesothelial extracellular matrix degradation and regulates transcription	Therapeutic targets	(50)
KRT5、KRT7	SKOV3	Up-regulated	Promotes adhesion of cancer cells	Prognostic marker, therapeutic targets	(51)
DLX-1	HEK293, A2780cp, OVCA433, OVC1021, SKOV3	Up-regulated	Regulates the TGF-β1/SMAD4 signaling pathway to promote cell migration and invasion	Therapeutic targets	(52)
VEGF	/	Up-regulated	Induces tumor angiogenesis	Therapeutic targets	(53, 54)
CCNF	A2780, SKOV3, OVCAR3, HEY, HEK293T	Up-regulated	Regulate the stability of proteins involved in the cell cycle and genome stability	Prognostic marker, therapeutic targets	(49)
KIF20A	A2780, SKOV3, OVCAR3, HEY, HEK293T	Up-regulated	Accumulates in mitotic cells, involved in mitosis and chromosome transport	Prognostic marker, therapeutic targets	(49, 55)
PDGF	/	Up-regulated	Promotes angiogenesis	Therapeutic targets	(56, 57)

PLK1 and KIF20A are involved in cytoplasmic segregation during mitosis and contribute to cancer cell proliferation. As downstream genes of FOXM1, they are upregulated by FOXM1 (45, 49). In one study, Renata A. Tassi et al. reported that silencing FOXM1 in two epithelial ovarian cancer (EOC) cell lines—clear cell (EOC-CC1) and serous (OSPC2)—led to decreased expression of cell cycle-related genes, such as CCNB1 and CDC25B (41). Additionally, SKP2, a key subunit of the ubiquitin ligase complex SCF, can be directly bound by FOXM1 to increase its transcription. This promotes the degradation of p27^kip1^ by SCF, accelerating cell cycle progression (48). CCNF stabilizes proteins involved in cell cycle progression and genome stability, and its expression is increased by FOXM1 overexpression (49). Certain miRNAs also regulate FOXM1 expression and influence OC cell proliferation (58–60). For example, In 92 OC patients, miR-506 overexpression reduces FOXM1 through the CDK4/CDK6-FOXM1 pathway (58), while miR-370 inhibits FOXM1, counteracting its effects on proliferation, migration, and epithelial-mesenchymal transition (EMT) (60). Additionally, FOXM1 also regulates metabolic pathways to affect OC cell proliferation, upregulating key glycolytic enzymes including GLUT1 and HK2 to promote metabolic reprogramming (61). Notably, although FOXM1 is thought to be a regulator of cell cycle genes, little is known about the specific effects of its isoforms on downstream targets. Furthermore, the role of FOXM1 in metabolic re-editing may be masked by co-existing mutations, such as KRAS mutations, and subtype-specific analyses are needed to reconcile the different findings (62).

The metastatic process in tumors involves intricate stages including local tumor cell invasion, angiogenesis, formation of metastatic nodules, and eventual colonization at distant sites (63). Both tumor growth and metastasis depend on angiogenesis, which supplies necessary oxygen and nutrients (64). In OC cases, high FOXM1 expression positively correlates with increased microvessel density. As a TF, FOXM1 regulates angiogenesis by upregulating VEGF promoter activity, elevating VEGF mRNA and protein levels, and thus promoting angiogenesis, tumor proliferation, and invasion (65). Additionally, platelet-derived growth factor (PDGF) plays a significant role in the angiogenesis of OC tissues (66).

Downstream target genes of FOXM1 have been identified that affect cancer cell invasion and migration. For example, Zhang et al. found that FOXM1 expression was positively correlated with KRT5 and KRT7 expression, and knockdown of these genes reduced the migration of cancer cells (51). Matrix metalloproteinases (MMPs), proteases that degrade extracellular matrix (ECM) proteins, promote OC cell metastasis via ECM remodeling, EMT, and transcriptional regulation (50). High expression of DLX1 is strongly associated with advanced OC development, and FOXM1 can bind to the DLX1 promoter region, activating DLX1 expression. This enhances cancer cell migration and invasion through TGF-β1/SMAD4 signaling (52). The regulation of downstream target gene expression by FOXM1 promotes cancer cell proliferation and metastatic implantation via multiple pathways, further highlighting its critical role in OC development. Further studies may reveal other FOXM1 signaling pathways associated with OC formation, growth and metastasis.

### 3.2 FOXM1 promotes cancer stemness

Ovarian cancer stem cells (OCSCs) are noted for their robust self-renewal and adaptability. They significantly contribute to the persistence, low remission rates, high recurrence, and adverse outcomes associated with OC (66, 67). Prior research indicates that OCSCs endure standard chemotherapy and exhibit pronounced metastatic capacities. More importantly, metastatic OC often shows heightened resistance to chemotherapeutic agents, diminishing the efficacy of standard treatment protocols (68). In this context, FOXM1 emerges as a vital component for OC stem cells, playing a critical role in tumorigenesis.

Some studies have found that OCSCs, upon contact with the peritoneal tumor microenvironment (TME), activate the cell cycle pathway, increasing the self-renewal rate of cancer cells. This interaction also activated the FAK-YAP pathway, and induced FOXM1 expression. Interference with FOXM1 inhibited OCSC survival (69). Additionally, FOXM1 regulates the stemness of OC cells and promotes tumor progression by interacting with the WNT/β-catenin signaling pathway (70). Chemotherapy resistance may indicate that cancer cells possess stem cell-like properties. A previous study reported an increase in the average expression of cancer cell markers (CD44, ALDH1A1, and CD133) in recurrent OC samples compared to primary OC samples from the same patients. Notably, CD133 was almost always elevated in recurrent samples, with the proportion of positive cells more than doubling in 58% of the samples (71).

Ning et al. showed that OCSC markers ALDH, CD133, and CD144 were highly expressed in OC cells. DFOG downregulated the expression of OCSC markers and FOXM1, inhibiting cancer cell self-renewal. However, overexpression of FOXM1 reversed this effect, enhancing the self-renewal capacity of OCSCs and promoting cancer cell stemness, leading to more severe disease (67). In addition to OC, cancer cell stemness has been associated with FOXM1 in other cancers, including breast, colon, prostate, lung, and endometrial cancers (72–76). These findings further suggest a strong correlation between FOXM1 and OCSCs, indicating that FOXM1 may serve as an important marker for evaluating the treatment and prognosis of OC patients.

## 4 Clinical transformation

### 4.1 The potential of FOXM1 as a biomarker for OC

Early tumor diagnosis depends largely on biomarker testing, which is crucial for personalized medicine (77). Despite the discovery of thousands of biomarkers in recent years, only a few are directly applicable in clinical practice (78). OC is a complex disease with varying cancer cell morphologies and biological behaviors (79). The detection of specific biomarkers can facilitate early diagnosis and prompt medical intervention (3). However, current tumor markers have limitations, particularly in the early stages of OC (3). Thus, there is an urgent need to identify more reliable biomarkers for this disease.

Studies have shown that FOXM1 is abnormally expressed in various cancer cells and can be used as a biomarker for cancer diagnosis and treatment (80–83). A comprehensive meta-analysis by Andrew J. Gentles et al., involving around 18,000 tumor samples across 39 different cancers, highlighted FOXM1 as a critical prognostic marker indicative of poor outcomes across a broad cancer spectrum (84). Various studies have aligned FOXM1 overexpression with heightened tumor grade, stage, and increased disease severity (39, 41, 43, 85). In a specific investigation of 90 EOC patients, encompassing 50 cases of high-grade serous carcinoma (HGSC), 14 of clear cell-like EOC, and 26 of endometrioid EOC, elevated FOXM1 levels were significantly prevalent in plasmacytoid EOC and correlated with advanced FIGO stages (P = 0.004) (41).

Carter J. Barger et al. also reported significantly higher FOXM1 expression in patients with advanced and high-grade OC, suggesting that FOXM1 may serve as an independent marker of poor prognosis (43). While most studies have indicated a correlation between FOXM1 expression and the staging and grading of OC, some studies have reported no significant association. For example, Ning et al. found that FOXM1 expression is related to lymph node metastasis, but not significantly associated with FIGO staging (P = 0.127) or grading (P = 0.298) (40). These conflicting results may stem from various factors, such as differences in antibody specificity, sample size variations, or inconsistencies in study methodology. Additionally, biological heterogeneity among OC subtypes could also account for the inconsistencies. These observations underscore the urgent need for large-scale studies to address methodological discrepancies in current research and to probe the underlying causes of the contradictory findings on FOXM1’s role in cancer prognosis.

The expression of FOXM1 is significantly linked with survival outcomes in OC patients. Ning and colleagues reported that higher FOXM1 levels are associated with poorer prognoses and reduced survival rates (40). Additionally, Zhang and his team observed that patients with elevated FOXM1 expression experienced a higher rate of recurrence during progression-free survival (PFS) and exhibited trends towards shorter overall survival (OS). Additionally, their study showed that FOXM1 protein improved the accuracy of OC recurrence prediction (86). In a meta-analysis of 23 studies, FOXM1 overexpression was associated with 3-year OS (OR = 3.30, 95% CI = 2.56 to 4.25, P < 0.00001), 5-year OS (OR = 3.35, 95% CI = 2.64 to 4.26, P < 0.00001), and 10-year OS (OR = 5.24, 95% CI = 2.61 to 10.52, P < 0.00001). It was also linked to worse OS in most solid tumors (85). Thus, FOXM1 holds significant potential as a biomarker for diagnosing and determining the prognosis of OC. Targeting FOXM1 could offer a promising therapeutic approach.

### 4.2 Chemotherapy resistance and targeted therapy in OC

**Figure 3:**
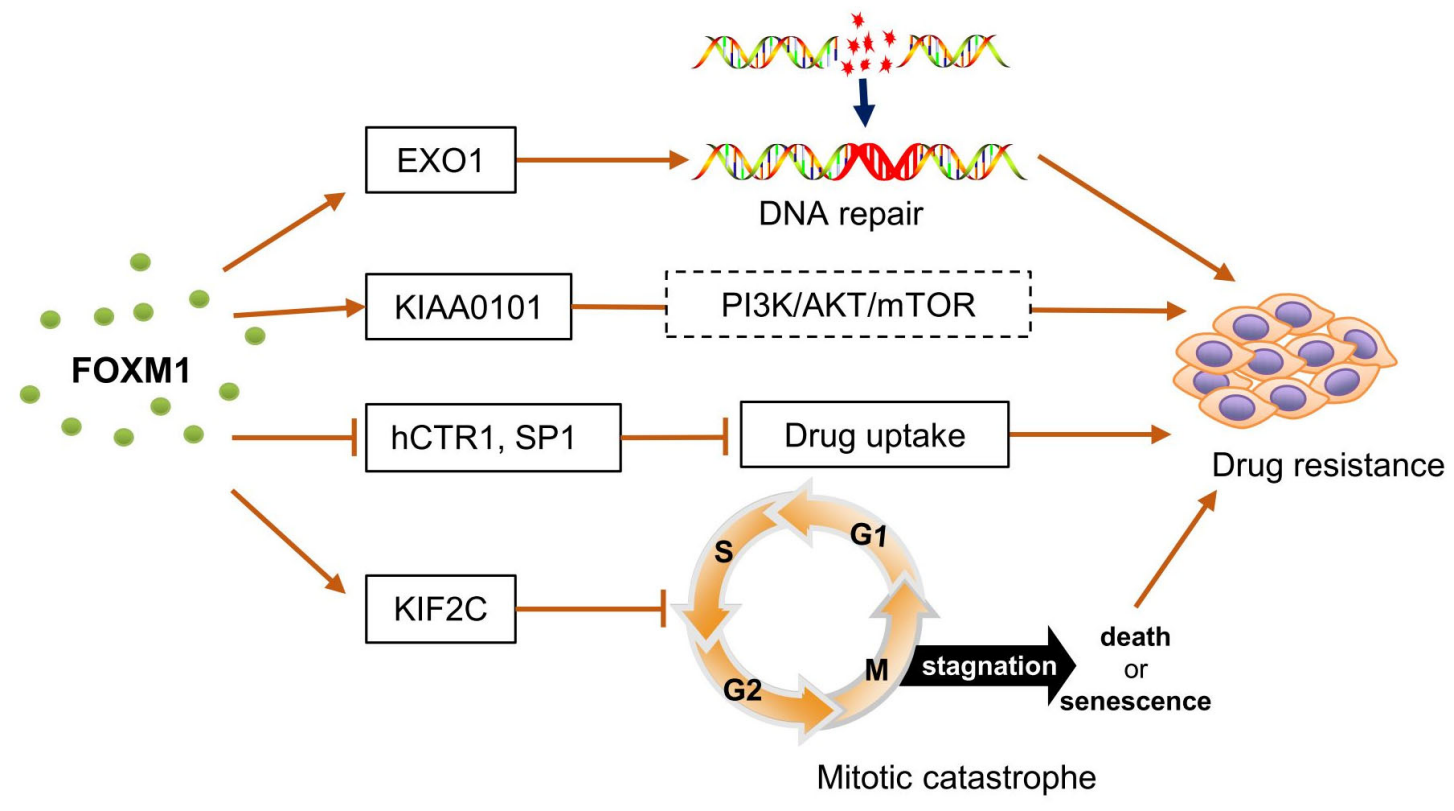
FOXM1 involved in chemoresistance in ovarian cancer. FOXM1 targeted and up-regulated EXO1 expression, which promoted DNA damage repair and led to cisplatin resistance in cancer cells.FOXM1 activated the expression of KIAA0101, which acted through the PI3K/AKT/mTOR signaling pathway, enhancing the viability of cancer cells and promoting the development of chemotherapy resistance. FOXM1 inhibits the expression of hCTR1 and SP1, which prevents the entry of cisplatin into ovarian cancer cells and reduces the sensitivity of ovarian cancer to cisplatin. FOXM1 inhibited mitotic catastrophe by up-regulating the expression of KIF2C, enabling cancer cells to survive and proliferate under chemotherapeutic stress. hCTR1, a transmembrane transporter protein that allows cisplatin to cross the membrane barrier into cells. SP1, transcription factor that induces the expression of hCTR1. Mitotic catastrophe, a tumor suppressor mechanism that detects mitotic errors and subsequently drives cells to an irreversible anti-proliferative end, death or senescence.

Chemotherapy remains a primary therapeutic approach for various tumors, significantly reducing cancer mortality (87). Nonetheless, resistance to chemotherapy constitutes a major challenge in OC treatment, being a primary cause of cancer fatalities among women (88). Chemoresistant OC cells exhibit stronger cancer stemness, invasive abilities, and metastatic potential (70). In recent years, the role of FOXM1 in chemoresistance has gained significant attention (7, 38) (**Figure 3**). Zhou et al. found that FOXM1 targets EXO1, a downstream gene related to DNA repair, and promotes DNA repair, leading to cisplatin resistance in cancer cells. Downregulation of FOXM1 increases the sensitivity of OC cells to cisplatin (38).

In other cancers, FOXM1 has also been shown to increase drug resistance by regulating downstream DNA repair targets such as RAD51 (89), NBS1 (90), BRIP1 (91), and BRCA2 (92). Additionally, FOXM1 activates the expression of KIAA0101, which blocks cisplatin-induced apoptosis and autophagy in OC cells through the PI3K/AKT/mTOR pathway, reducing cisplatin sensitivity (93). KIF2C, identified as a target gene of FOXM1, shows a similar expression pattern to FOXM1. FOXM1 blocks mitotic catastrophe in OC cells, thereby increasing paclitaxel resistance, a process potentially mediated by KIF2C (94).

Chiu et al. found that the WNT/β-CATENIN pathway induces FOXM1 expression, which inhibits the expression of human copper transporter protein 1 (hCTR1) and SP1, preventing cisplatin uptake in OC cells and leading to cisplatin resistance (70). These findings suggest a strong association between FOXM1 and drug resistance in OC, but there are still significant gaps in understanding the exact mechanisms by which FOXM1 regulates resistance. Particularly, FOXM1b and FOXM1c show high activity in a wide range of cancer types and especially play important roles in the proliferation and metastasis of tumor cells (95, 96). However, studies on FOXM1 subtypes in OC remain limited. Given the distinct roles of different FOXM1 isoforms in cell biology, developing specific inhibitors against these isoforms may offer greater targeted therapeutic potential and efficacy, potentially providing new insights for individualized OC treatment.

FOXM1 is highly expressed in OC cells, leading to chemotherapy resistance. This provides a strong rationale for targeting FOXM1 inhibition as a treatment strategy for OC. Two primary methods exist for inhibiting FOXM1: 1) inhibiting or activating upstream pathways of FOXM1, thereby indirectly affecting its activity, and 2) directly targeting and inhibiting FOXM1 (**Table 2**). Currently, few FOXM1 inhibitors are used in the clinic, and OC frequently develops drug resistance, making research into both indirect and direct FOXM1 inhibitors highly valuable.

**Table 2:** Compounds that target FOXM1 for inhibition of expression.

First author	Year	FOXM1 Inhibitors	Description	Action mechanism	Refs
Targeting FOXM1 upstream pathway					
Noack	2018	BI6727	a small molecule inhibitor of PLK1	Blocks FOXM1 activation via Inhibit of PLK1 kinase	(97)
Momeny	2017	Dacomitinib	a pan-ErbB receptor inhibitor	Blocks the PLK1-FOXM1 signalling pathway and its downstream targets Aurora kinase B and survivin	(98)
Li	2019	DADS	a volatile component of garlic oil	Suppress FOXM1 by up-regulating miR-134 expression	(27)
Targeting FOXM11 directly					
Radhakrishnan	2006	Siomycin A	a thiazole antibiotic	Prevents FOXM1 phosphorylation and down-regulates its mRNA and protein levels	(99)
Westhoff	2017	Thiostrepton	a thiazole antibiotic	Target FOXM1 and then downregulate the expression of FOXM1 mRNA and its downstream targets	(100)
Ning	2014	DFOG	a novel synthetic genistein analogue	Direct inactivating FOXM1	(67)
Ketola	2017	Monensin	a novel FOXM1-binding agent	Silence the expression of FOXM1 and its signalling pathway members	(101)
Gao	2022	XST-20	ethylene glycol phenyl aminoethyl ether derivatives	Binds to the DNA binding domain of FOXM1 and inhibits its transcriptional activity	(102)
Liu	2024	NB-73, NB-115	a novel class of FOXM1 inhibitors possessing a 1,1-diarylethylene core structure	Binds directly to FOXM1 compounds and promotes their degradation	(103)
Gormally	2014	FDI-6	3-amino-N-(4-fluorophenyl)-6-(thiophen-2-yl)-4-(trifluoromethyl)thieno[2,3-b]pyridine-2-carboxamide TFA	FDI-6 precludes binding of FOXM1 to consensus sequence DNA targets and broadly inhibits transcription of FOXM1-activated genes	(104)
Gartel	2010	Bortezomib, MG115, MG132	a proteasome inhibitor	Decrease mRNA and protein levels of FOXM1 and its target genes	(105)
Shukla	2019	RCM-1	non-toxic inhibitor of FOXM1	Disrupts the FOXM1-β-catenin interaction and inhibits the nuclear localisation of FOXM1 protein, causing reduced FOXM1 stability	(106)
Andrikopoulou	2o21	Q1, I-BET151	bromodomain and extra-terminal domain (BET) inhibitor	Down-regulation of the expression of FOXM1 and its downstream signaling pathways	(107)

FOXM1 expression is regulated by upstream signaling pathways, including MAPK/ERK, PLK1, and PI3K. Noack et al. demonstrated that the PLK1 inhibitor BI6727 reduced the viability of CCNE1-expanded OC cells, increased their sensitivity to paclitaxel, and induced apoptosis in cancer cells when combined with paclitaxel. Importantly, they suggested that PLK1 inhibitors may indirectly affect FOXM1 activity (97). This is because PLK1 is an upstream kinase that is essential for FOXM1 phosphorylation and activation based on the putative consensus phosphorylation sites (16, 97). This suggests a potential mechanistic link between PLK1 inhibitors and FOXM1 expression and activity, although further experiments are needed to fully elaborate this relationship. Dacomitinib can enhance OC cell sensitivity to cisplatin by inhibiting the ErbB receptor, reducing the expression of phosphorylated PLK1, and inhibiting FOXM1 activity (98). In osteosarcoma, diallyl disulfide (DADS) inhibits FOXM1 expression by activating miR-134, an upstream regulator of FOXM1, thereby reducing cancer cell proliferation and invasion (27).

Many compounds have been reported to directly inhibit FOXM1 expression. These include siomycin A (99), thiostrepton (100), DFOG (67), monensin (101), XST-20 (102), NB compounds (103), FDI-6 (104), Bortezomib (105), and RCM-1 (106), which exert their effects by regulating various biological processes involved in cancer cell development, including proliferation, migration, invasion, and apoptosis. Siomycin A and thiostrepton, thiazole antibiotics, were first reported as FOXM1 inhibitors, with thiostrepton being the most commonly used (99, 100). It targets FOXM1 to reduce mRNA expression and its downstream targets, leading to OC cell death. When combined with paclitaxel and cisplatin, thiostrepton may offer a novel approach for treating chemotherapy-resistant OC (100).

Siomycin A disrupts FOXM1’s transcriptional activity by impeding its phosphorylation, curtails anchorage-independent cellular growth in soft agar assays, and selectively induces apoptosis in transformed cells while sparing normal cells, making it a potential candidate for anticancer therapy (99). Genistein (4’, 5, 7-trihydroxyisoflavone; GEN) has been proven to suppress the proliferative capabilities of breast cancer stem cells (108). Additionally, Ning and colleagues have shown that DFOG effectively curtails the enhanced self-renewal abilities of cancer stem cells induced by abnormally elevated FOXM1 expression in OC cells (67).

In a prostate cancer study, monensin was found to bind to the DNA-binding domain (DBD) of FOXM1, reducing its interaction with downstream target genes such as PLK1 and CDC25B, thereby exerting an anti-cancer effect (101). NB compounds (NB-73, NB-115) promote FOXM1 protein degradation and inhibit the expression of target genes. These compounds show synergistic effects with carboplatin in high-grade serous OC (HGSOC) cells, potentially enhancing therapeutic efficacy (103). Unlike NB compounds, RCM-1 reduces tumor growth by disrupting the interaction between FOXM1 and β-catenin (106). In addition, studies targeting BET (Bromodomain and Extra-Terminal domain) inhibitors in OC treatment continue to intensify (107). In particular, BET inhibitors such as JQ1 or I-BET151 have been shown to be able to effectively inhibit the proliferation and migration of OC cells by down-regulating the expression of FOXM1 and its downstream pathways, leading to therapeutic effects (109, 110).

These compounds that inhibit FOXM1 expression have demonstrated anti-tumor effects, with some also enhancing cancer cell sensitivity to drugs. This suggests that targeted inhibition of FOXM1 could offer a novel strategy for OC treatment. However, further studies are needed to explore the activation of other pathways and determine whether the observed anti-tumor effects are attributable to FOXM1 inhibition. Additionally, the precise mechanisms by which these compounds inhibit FOXM1 expression in OC require further investigation.

### 4.3 Clinical translational potential and challenges of FOXM1 inhibitors

Currently, most inhibitors remain in preclinical research, mainly assessing safety and efficacy (107, 111). Only a few BET inhibitors, such as JQ1, have entered early clinical trials in OC (107). Thiostrepton has shown efficacy in preclinical trials in OC and other tumors but lacks widespread clinical approval (111–113). Despite promising antitumor activity *in vitro* and animal models, FOXM1 inhibitors’ clinical application is hindered by poor pharmacokinetic properties and potential toxicity, with bioavailability, half-life, and tissue distribution issues affecting efficacy (114, 115). Therefore, optimizing the chemical structure of inhibitors to enhance their bioavailability and reduce their toxicity is the focus of current research.

To boost FOXM1 inhibitors’ clinical efficacy, researchers are exploring combination therapies (107). For example, combining the FOXM1 inhibitor thiostrepton with PARP inhibitors yields a synergistic effect (111). Combining FOXM1 inhibitors with immune checkpoint inhibitors also shows great therapeutic potential. PD-1/PD-L1 antibodies, which alleviate immunosuppression in the tumor microenvironment, enhance T-cell antitumor activity. Thiostrepton, in turn, directly inhibits tumor cell proliferation and metastasis, and their combination may produce a synergistic therapeutic effect (116). In melanoma models, combating CTLA-4 inhibitors with I-BET151 also shows a trend toward enhanced anti-tumor activity (117). Combining FOXM1 inhibitors, particularly with immune checkpoint inhibitors, is expected to open new tumor therapy avenues.

## 5 Conclusions and perspectives

In conclusion, FOXM1 plays a crucial role in the development of OC. It possesses the distinct characteristics of a biomarker, making it valuable for predicting survival. Although its clinical utility has been demonstrated across various cancers, its role in immune escape mechanisms remains largely unexplored, including its interactions with immune checkpoint molecules and its impact on the activity and function of immune cells. The unique contributions of different FOXM1 subtypes to immune escape are also poorly understood. Tumor spatial heterogeneity has been widely recognized, and FOXM1 may play an important role in this process; however, its expression patterns across different tumor regions and its impact on the spatial organization of the tumor microenvironment remain unclear.

Currently, the application of FOXM1 inhibitors in OC treatment is limited. Before FOXM1 inhibitors can be used in clinical practice, further in-depth studies on their anti-tumor effects and thorough evaluations of their toxicity are needed. The development of novel and effective FOXM1-targeted therapies remains challenging. A deep understanding of FOXM1’s regulatory role, especially in immune evasion and tumor microenvironment heterogeneity, will offer fresh insights into OC research and may unveil new therapeutic avenues.
